# Internal limiting membrane insertion technique combined with nerve growth factor injection for large macular hole

**DOI:** 10.1186/s12886-019-1258-z

**Published:** 2019-12-10

**Authors:** Luyi Zhang, Xiaoxia Li, Xiaoli Yang, Yu Shen, Miaoqin Wu

**Affiliations:** Department of Ophthalmology. Zhejiang Provincial People’s Hospital, People’s Hospital of Hangzhou Medical College, 158 Shangtang Road, Hangzhou, 310014 Zhejiang China

**Keywords:** Nerve growth factor, Macular hole, Internal limiting membrane insertion, Photoreceptor layers recovery

## Abstract

**Background:**

The study was proposed to determine whether nerve growth factor (NGF) combined with an internal limiting membrane (ILM) insertion was effective in the large idiopathic full-thickness macular hole (iFTMH) therapy.

**Methods:**

A subset of 18 eyes (July 2015–October 2017) diagnosed as the large iFTMH were enrolled in this study. The subjects were treated using ILM insertion technique alone (ILM group) or ILM combined with NGF injection (NGF group) and the follow-up period was 6 months. Macular hole closure rates, best-corrected visual acuity (BCVA, improvements using ETDRS), and optical coherence tomography (OCT) findings were analyzed at 1st, 3rd, and 6th months postoperatively.

**Results:**

We found that macular holes in both groups fully closed. In comparison to ILM insertion group, the NGF group had better BCVA at the 3rd month (48.00 ± 2.392 vs 58.22 ± 2.957, 95% confidence interval (CI): 2.159 to 18.29). The mean external limiting membrane (ELM, 422.2 ± 96 vs 674.9 ± 103.6, 95% CI: − 47.26 to 552.8) and ellipsoid zone (EZ, 496.7 ± 101.6 vs 766.7 ± 111.8, 95% CI: − 50.29 to 590.4) defects were significantly smaller in the NGF group at the 6th month in the follow-up examination. Complete recovery of ELM and EZ was observed in the NGF group in one eye of a patient and two eyes of two patients, respectively. In comparison, one eye’s ELM and another eye’s EZ were completely recovered in the ILM insertion group.

**Conclusion:**

Our results indicated that ILM insertion with NGF injection might be an effective technique for the initial surgical treatment of eyes with large MHs. The proposed approach yielded better recovery of the photoreceptor layers and consequently might have superior postoperative visual acuity.

**Trial registration:**

chiCTR1900021711. Retrospectively registered 5 March 2019.

## Background

A full-thickness macular hole (FTMH) is a complete interruption of neurosensory retina tissue at the fovea that causes deterioration of central vision [1]. The incidence of macular hole (MH) is 0.1–0.3% in the general population; 11.7% of MHs are bilateral, most of which are idiopathic [2, 3]. FTMHs were considered to be untreatable prior to the remarkable achievement of Kelly and Wendel, who used pars plana vitrectomy (PPV) to treat FTMHs for the first time. Currently, combined PPV with or without internal limiting membrane (ILM) peeling and gas tamponade is the standard surgical strategy for FTMH [4, 5]; Clinically, using this method, the success rate of anatomical closure is 85–90% [6–9]. However, in patients with large MHs (diameter > 400 μm), the surgical outcomes are usually very poor; 44% of holes do not close after the first surgery and 19–39% are flat-to-open after operation [10]. To improve the closure rate in such cases, two remarkable novel surgical techniques have been developed: the inverted flap technique (primary operation method for iFTMH treatment) and the free-flap technique [11, 12].

Several reports have supported the efficacy of both techniques, but some authors reported the absence of visual recovery and persistent defects in the outer photoreceptor segments [13, 14]. Although a large proportion of MHs fully heal after surgery, the best-corrected visual acuity (BCVA) remains unsatisfactory. For example, optical coherence tomography (OCT) often reveals residual attachment of the ellipsoid zone (EZ) and external limiting membrane (ELM) at the junction between inner and outer segments of the photoreceptors (IS/OS junction) and the junction between inner segments and ELM; while integrity of these layers is essential to ensure good postoperative visual acuity after surgery [15, 16].

Nerve growth factor (NGF), a classical neuroprotective factor, increases vascular endothelial growth factor (VEGF) expression and promotes cell proliferation [17]. In China, NGF has been approved by National Medical Products Administration(NMPA) for treatment of optic nerve injury due to these characteristics. NGF, produced by Müller cells, plays a critical role in retinal neovascularization [18], influencing ILM metabolism. Previously, we have found the promotion effects of NGF, ILM, and NGF plus the ILM on Müller cell proliferation in vitro, which provided novel insights into the association between the ILM and primary Müller cells in co-culture. We also examined the underlying regulatory mechanism involved in NGF- and ILM-mediated cell growth, using inhibitors of some key effectors [19]. Overall, we found that NGF clearly promoted Müller cell proliferation in co-culture with the ILM; thus, NGF may serve as a neuroprotective agent during MH treatment.

In this study, though both ILM and ILM plus NGF promoted the complete healing of MH, NGF group had better BCVA and good recovery of ELM and EZ compared to ILM insertion alone group, which might provide a new view and reference for clinical prevention and treatment for FTMHs.

## Methods

### Patients

This is a prospective study that enrolled the subjects with surgical iFTMH diagnosed at Zhejiang Provincial People’s Hospital. Firstly, 7 Chinese patients who had iFTMH between September 2016 and October 2017 were enrolled. The patients underwent phacoemulsification, 23-G PPV, ILM flap insertion with NGF intravitreal injection and placement of a C3F8 tamponade (the NGF group). The inclusion criteria were age over 40 years, mild cataracts, the diameter of the MH > 400 μm, and the absence of other ocular diseases such as pre-existing ocular diseases or a history of retinal surgery, especially PPV and scleral buckle. Each patient was informed about the risks and benefits of surgery. Then we retrospectively enrolled 9 patients who had diagnosed as iFTMH between July 2015 and August 2016 in Zhejiang Provincial People’s Hospital. These patients who had underwent phacoemulsification, 23-G PPV with ILM peeling, ILM flap insertion, and placement of a C3F8 tamponade were served as the control group (the insertion group, control). The inclusion criteria were the same as the NGF group. This study adhered to all relevant tenets of the Declaration of Helsinki. The Institutional Review Board/Ethics Committee of Zhejiang Provincial People’s Hospital approved the study protocol. Subjects were provided a written informed consent in accordance with the guidelines of Zhejiang Provincial People’s Hospital.

### Surgical technique

All surgeries were performed by the same experienced surgeon (M.Q. Wu). General anesthesia was performed for all cases. Phacoemulsification was performed on all phakic eyes prior to PPV and the intraocular lens was implanted at the end of the surgery. The core and peripheral vitreous were excised using a non-contact wide-angle viewing system (BIOM; Oculus Inc., Wetzlar, Germany) and a conventional 23-gauge three-port procedure. Indocyanine green (0.025% w/v dye solution) was used to stain the ILM for 1.5 min. Approximately two-disc diameters of ILM around the MH were peeled, and 90–120° (1–1.5 quadrants) of the ILM flap (about 0.5 of the disc area) was left attached to the edge of the MH. The flap was trimmed with a vitreous cutter if needed, and then smoothly inserted into the MH to cover the entire base. After intraocular lens implantation, fluid-gas exchange was performed and 0.8 mL of 100% C3F8 was injected into the vitreous cavity (final ratio was 14%). In the NGF injection group, one drop (0.06 mL) of 6 μg/mL NGF which was isolated from mice submandibular glands was placed in the macular fovea prior to gas tamponade (Additional file 1). NGF for injection is from commercialized medicinal products which guaranteed the sterility, safety and efficacy of NGF (Mouse Nerve growth factor for injection, Staidson, Beijing, China). Patients remained face-up for 6 h after surgery in the hospital to allow the position of the ILM flap and the absorption of NGF. Then patients were told to remain face-down for 7 days postoperatively.


**Additional file 1** Surgery. a video for our new surgical procedure. (MP4 17895 kb)


### Physical examination

After the operation, all patients underwent a complete ophthalmological examination, including measurement of BCVA and intraocular pressure, indirect ophthalmoscopy, slit-lamp biomicroscopy, axial length measurement via partial coherence interferometry (IOL Master; Carl Zeiss Meditec, Dublin, CA, USA), and OCT (Cirrus HD-OCT; Carl Zeiss Meditec). BCVA was measured using an ETDRS chart to allow statistical analysis. OCT scans were obtained over a 9 × 9-mm^2^ area centered on the fovea; the scan density was 512 A-scans (horizontal) × 128 B-scans (vertical); hole diameters and the lengths of any defects in the EZ and ELM were measured. The defects were measured again after surgery using the embedded manual caliper function of the OCT platform. Hole sizes were calculated as the average of the vertical and horizontal diameters of the narrowest area. EZ and ELM defect sizes were calculated by the mean vertical and horizontal (discontinuous) lengths of hyper-reflective lines corresponding to these tissues. The foveal configuration was characterized as U-, V-, or W-shaped, or as open. The U-shape was similar to that of a healthy fovea, whereas the V-shape featured a steep contour with a thin foveal centralis. The W-shape was a closed but irregular contour that was not a U- or V-shaped [20].

### Statistical analyses

Between-group differences were compared using the t-test or chi-squared test. Data were presented according to three independent experiment and appeared as mean ± standard error of the mean and data analysis was based on GraphPad Prism 5.0 (GraphPad Software Inc., USA) software. A *P*-value < 0.05 was considered to reflect statistical significance.

## Results

A set of 18 eyes (12 right eyes and 6 left eyes) from 16 patients (2 males and 14 females) were included in our study. Among these subjects, 9 eyes from 9 patients (July 2015–August 2016) were in the ILM insertion group and 9 from 7 patients (September 2016–October 2017) in the NGF group. Subjects receiving different treatment were followed for 6 months. Before conducting the administration of NGF, the clinical data of subjects in ILM insertion group including BCVA and the ELM and EZ defect size were obtained from patient medical diaries and graphical clinical readings throughout the follow-up period and analyzed. After that, we enrolled another 9 eyes to determine the effect of ILM insertion plus NGF on large MHs. Similarly, the follow-up period was 6 months after surgery and the same clinical data were collected and analyzed. Among the 18 recruited eyes, all had large idiopathic MHs (minimum diameter > 400 μm and maximum diameter < 900 μm with average diameter of 593.1 ± 34.3 μm). All patients were phakic at baseline. The mean age of patients in the ILM insertion group was 63.7 ± 1.5 years, and it was 58.1 ± 3.3 years (95% CI: − 2.100 to 13.21) in the NGF group. No between-group difference in age, EZ or ELM defect size, optic axial length, cataract status, BCVA at baseline, or gender was observed (Table 1, chi-squared test). No patient had an open MH at the end of follow-up, showing 100% closure rate in both groups. All patients were pseudophakic at the end of surgery.

**Table 1:** Demographic and baseline clinical characteristics

	Insertion-only group Mean ± SEM	NGF group Mean ± SEM	*P*-value
Number of eyes	9	9	1.000
OD/OS	7/2	5/4	0.3100
Gender (M, F)	1, 8	1, 6	0.7000
Age (years)	63.67 ± 1.546	58.11 ± 3.264	0.0717
Axial length (mm)	23.27 ± 0.225	23.38 ± 0.498	0.4200
Cataracts (Stage I, Stage II)	4, 5	4, 5	1.000
BCVA (ETDRS letters)	41.89 ± 3.795	48.00 ± 2.392	0.0960
EZ defect size (μm)	1788 ± 151.7	1794 ± 123.3	0.4861
ELM defect size (μm)	1620 ± 110.8	1744 ± 114.2	0.2234

*BCVA* Best-corrected visual acuity, *EZ* Ellipsoid zone, *ELM* External limiting membrane

### Comparison between the ILM insertion versus NGF plus ILM insertion eyes

**Fig. 1:**
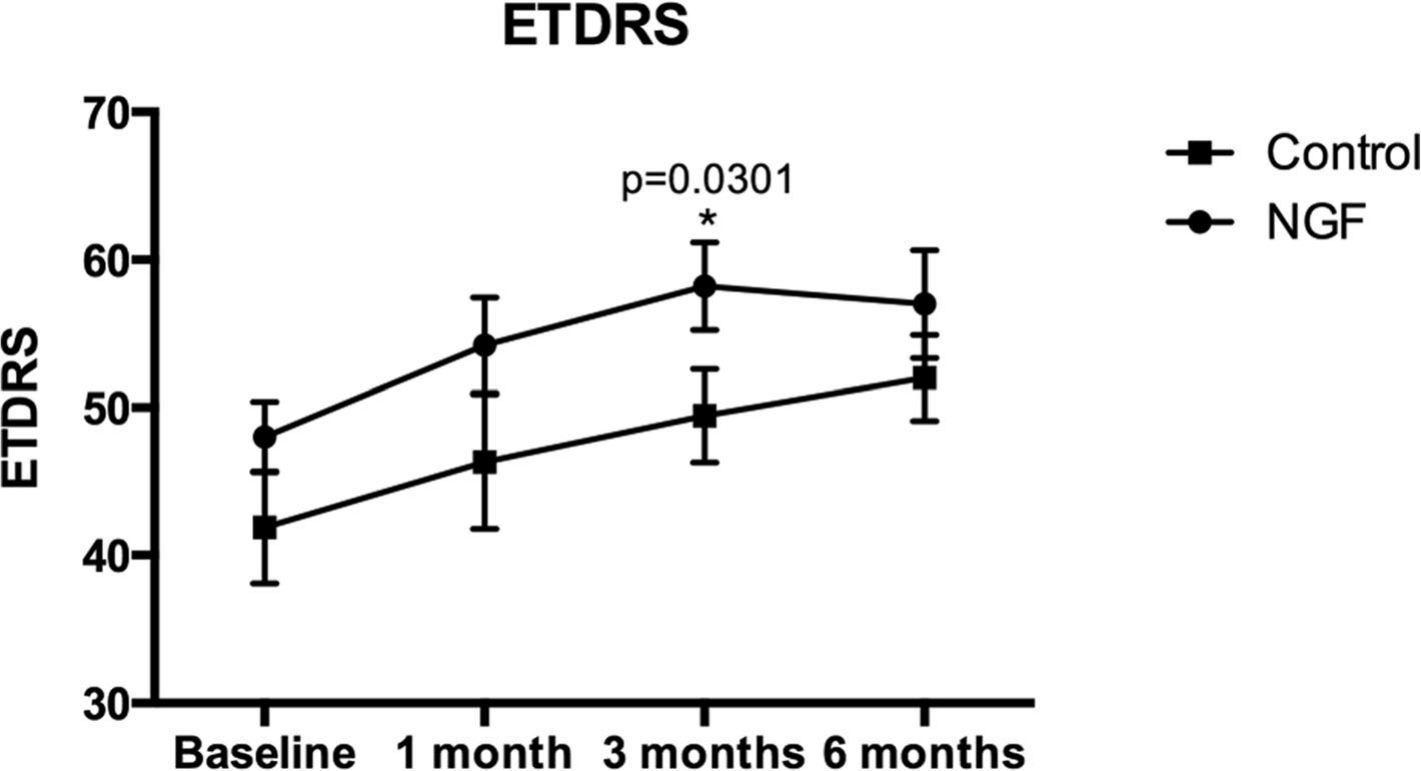
Changes in mean best-corrected visual acuity (BCVA; measured in terms of ETDRS letters to allow for statistical analysis) after macular hole (MH) surgery using the internal limiting membrane (ILM) insertion technique with nerve growth factor (NGF) injection (solid circles) or ILM insertion alone (solid squares). BCVA significantly improved at 3rd and 6th months after surgery in the NGF group, and in the insertion group after 6 months *(P* = 0.0081, 0.0276, and 0.0255, respectively, compared to baseline). However, the NGF group exhibited better recovery than the insertion group at 3rd month after surgery (*P* = 0.0301). The error bars are SEMs. ^a^*P* < 0.05

The BCVA of all patients were improved after surgery. The NGF group exhibited significant improvements in BCVA at both 3rd and 6th months (*P* = 0.0081, 95% CI: 2.159 to 18.29 and *P* = 0.0276, 95% CI: − 0.2269 to 18.23 compared to baseline, respectively, one-tailed unpaired t test), but not at 1st month postoperation. The insertion group exhibited significant improvement at 6th month (*P* = 0.0255, 95% CI: − 0.05218 to 20.27 compared to baseline, one-tailed unpaired t test). The NGF group exhibited a better BCVA at 3rd month than the insertion group (Fig. 1, *P* = 0.0301, 95% CI: − 17.01 to − 1.224, one-tailed unpaired t test); at other times, although statistical significance was not attained, the BCVA of the NGF group was better than that of the insertion group at 6th month.

**Fig. 2:**
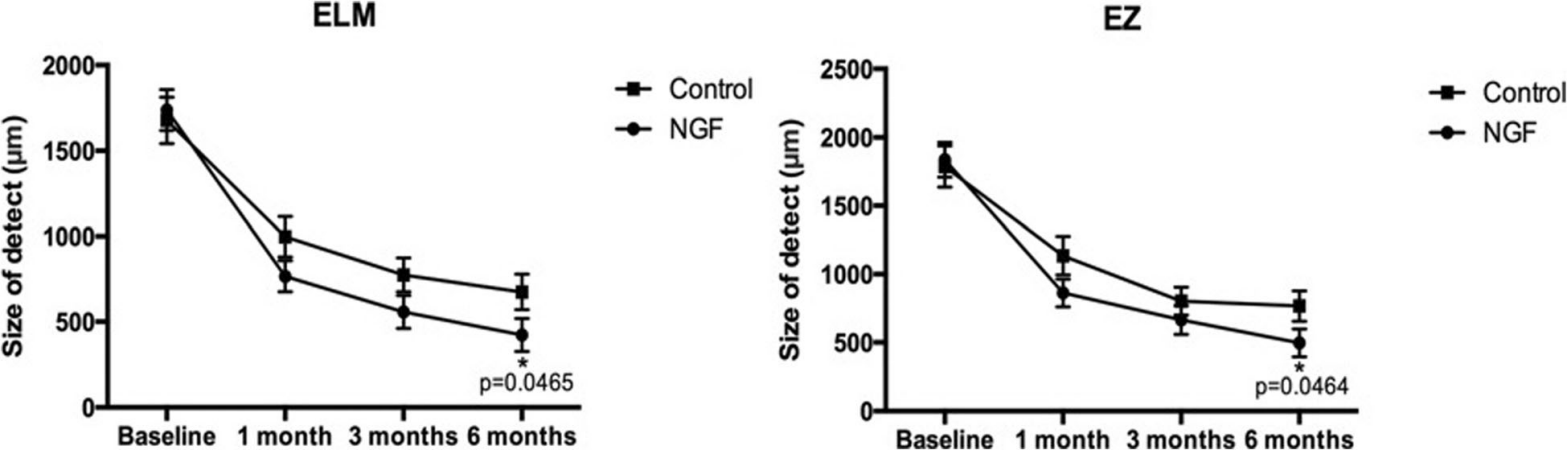
Changes in external limiting membrane (ELM) and ellipsoid zone (EZ) defects after MH surgery using the ILM insertion technique with NGF injection (solid circles) or ILM insertion alone (solid squares). The mean ELM and EZ defect sizes decreased significantly at 1st, 3rd, and 6th months after surgery in both groups (ELM: *P* < 0.0001, < 0.0001, and < 0.0001, =0.0008, < 0.0001, and < 0.0001, respectively, compared to baseline; EZ: *P* < 0.0001, < 0.0001, and < 0.0001, =0.0031, < 0.0001, and < 0.0001, respectively, compared to baseline). Defect resolution was significantly better in the NGF than the insertion group (*P* = 0.0465 and 0.0464 for ELM and EZ, respectively). The error bars indicate SEMs. *P < 0.05 for the comparison between the two groups

OCT revealed significant decreases in the sizes of ELM and EZ defects over time in both groups (Fig. 2) (ELM: *P* < 0.0001, < 0.0001, and < 0.0001, =0.0008, < 0.0001, and < 0.0001, compared to baseline; 95% CI: − 1292 to − 653.3, − 1508 to − 854.1, − 1643 to − 990.3, − 1063 to − 299.1, − 1260 to − 547.4 and − 1366 to − 641.8, respectively; EZ: *P* < 0.0001, < 0.0001, and < 0.0001, =0.0031, < 0.0001, and < 0.0001, compared to baseline, 95% CI: − 1317 to − 628.4, − 1521 to − 820.7, − 1684 to − 994.2, − 1091 to − 213.8, − 1372 to − 597.6 and − 1420 to − 621.2, one-tailed unpaired t test). At 6th month, the mean ELM defect size was 422.2 ± 96 μm in the NGF group and 674.9 ± 103.6 μm in the insertion group (95% CI: − 47.26 to 552.8), whereas the mean EZ defect sizes were 496.7 ± 101.6 and 766.7 ± 111.8 μm (95% CI: − 50.29 to 590.4), respectively. Significant improvements in EZ and ELM defects were observed in both groups (*P* < 0.0001 and < 0.0001 compared to baseline, respectively, as mentioned above). At 6th month postoperation, the mean sizes of ELM and EZ defects were significantly smaller in the NGF than the insertion group alone (*P* = 0.0465 and 0.0464, 95% CI: − 47.26 to 552.8 and − 50.29 to 590.4). Since the mean sizes of ELM and EZ defects at 1st and 3rd months postoperation were smaller in NGF group, there was no statistical significance between them (ELM: *P* = 0.0704 and 0.0685, 95% CI: − 85.12 to 548.0 and − 76.90 to 510.9; EZ: *P* = 0.0682 and 0.1804, 95% CI: − 95.64 to 639.2 and − 172.4 to 447.5, respectively, one-tailed unpaired t test). Two eyes in NGF group and one eye in insertion-only group exhibited complete ELM recovery; one eye in the NGF group and one eye in the insertion group exhibited complete EZ recovery at 6th month; no between-group differences were observed.

**Fig. 3:**
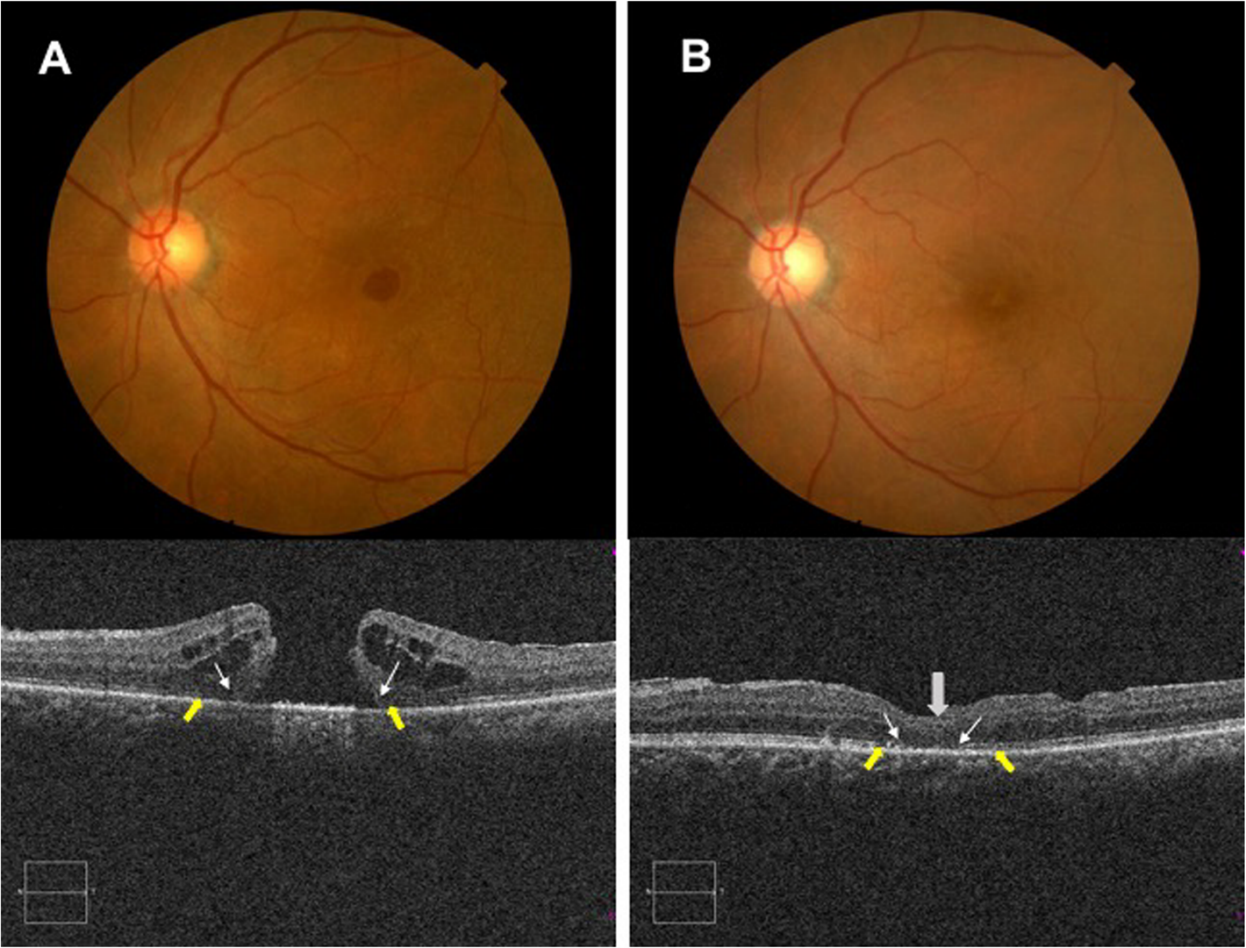
A representative case in the insertion group. **a** Baseline fundus photographs reveal a large, full-thickness macular hole (FTMH). Optical coherence tomography (OCT) reveals a large hole of minimum diameter 678.5 μm. The defects in the ELM (white arrowhead) and EZ (yellow arrowhead) measured 1779 μm and 1963.5 μm, respectively. **b** At 6th month after surgery, hole closure was evident on fundus photography. OCT showed that the fovea was filled with glial tissue (grey arrow) and that the closure was U-shaped. The ELM and EZ defects decreased to 565 and 812 μm, respectively. The BCVA improved from 39 to 48 letters

**Fig. 4:**
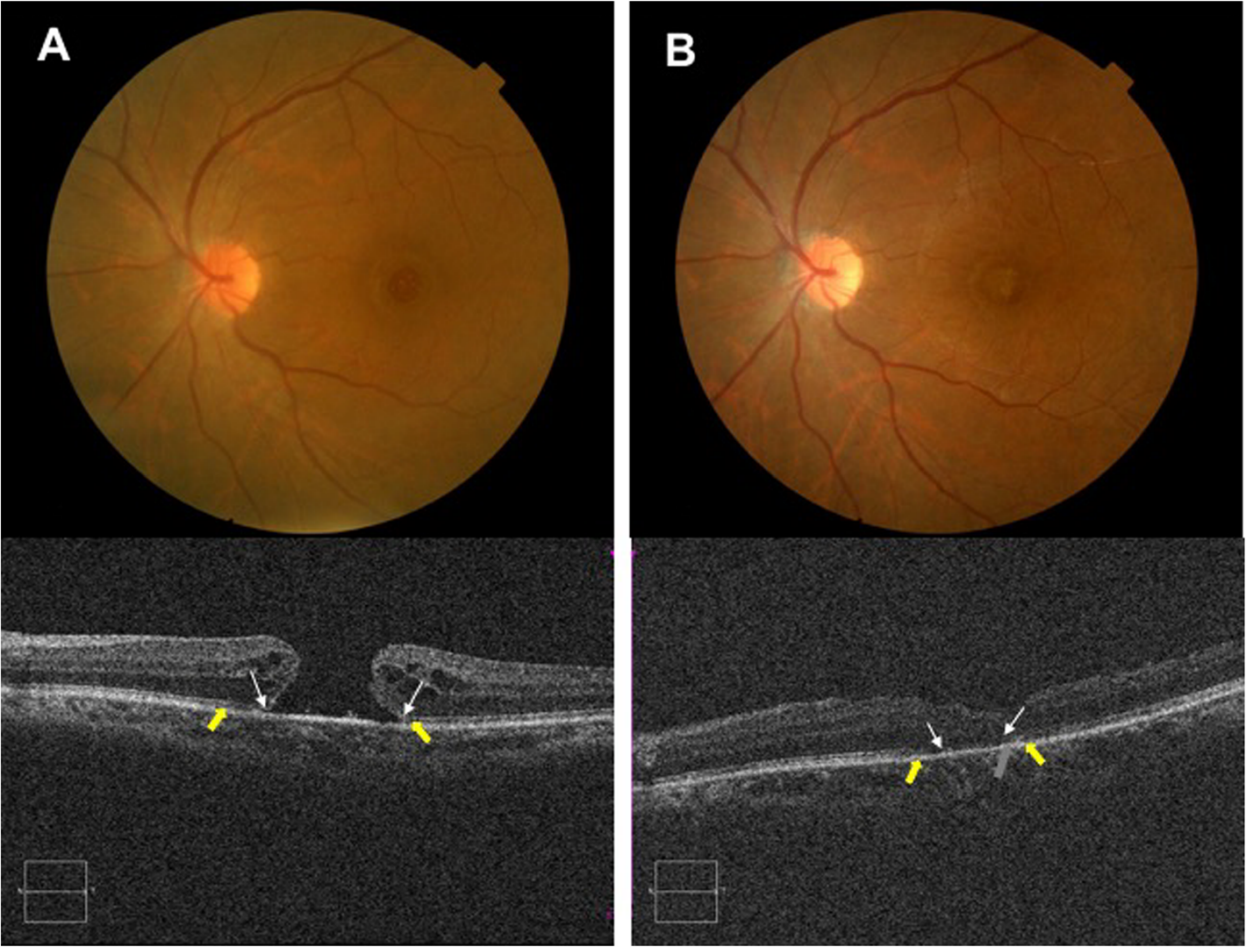
A representative case in the NGF group. **a** Baseline fundus photographs reveal a large FTMH. The OCT reveals a large hole of minimum diameter 711.5 μm. The defects in the ELM (white arrowhead) and EZ (yellow arrowhead) measured 1489 μm and 1789 μm, respectively. **b** At 6th month after surgery, hole closure was evident on fundus photography. OCT revealed that the closure was more like a V-shaped, a small part of glial tissue (grey arrow) could been found. The ELM and EZ defects decreased to 537 and 762 μm, respectively. The BCVA improved from 38 to 57 letters

All MHs closed (as confirmed by OCT) by 1 month after surgery and remained closed at 6th month (Figs. 3 and 4). At 6th month, five of nine foveae in NGF group were U-shaped (55.6%), three were V-shaped (33.3%), and one was W-shaped (11.1%). In the insertion-only group, three of nine foveae were U-shaped (33.3%), four were V-shaped (44.5%) and two were W-shaped (22.2%); there was no significant difference between the two groups (95% CI: − 11.49 to 274.8).

## Discussion

We showed that ILM insertion combined with NGF injection was effective at early period of postoperation for patients with large idiopathic MHs (minimum diameter > 400 μm). To the best of our knowledge, this is the first time that NGF has been used as a neuroprotective agent during MH treatment. Both surgical techniques produced excellent anatomical and functional outcomes throughout the entire follow-up time, without ILM sheet dislocation. OCT revealed the significant reductions in the sizes of EZ and ELM defects. The improvement of BCVA in the NGF group at 3rd month postoperation was better than that of the insertion-only group, as was photoreceptor layer re-establishment.

ILM peeling always aids the anatomical closure of MHs [21, 22], but the closure rate for large MHs after the first surgery with ILM peeling is not very high and usually results in poorer visual acuity. Moreover, large MHs always tend to be V- or W-shaped, or flat-to-open (a flat MH lacking retinal pigment epithelium) after closure, and are usually associated with persistent photoreceptor loss, retinal pigment epithelium defects, and foveal tissue loss, leading to a poor visual recovery [11, 23]. Several treatment modifications have been tested, including an inverted ILM flap technique [10] and autologous ILM transplantation [24]. The inverted flap may not attach to the hole margin either during or after surgery, as the flap loss using OCT scan after the surgery could be observed. While free ILM fragment transplantation may afford only a small degree of clearance of liquid and result in instability during gas-liquid exchange. However, all the above therapeutic strategy has low closure rate, insufficient photoreceptor layer re-establishment and consequently poorer visual acuity. In 2016, Andrew et al. described a modified technique that place the ILM directly into the MH, in any direction and a viscoelastic cap is used to improve flap retention [25]. Lai et al. also used blood clots to stabilize and seal ILM flaps within MHs during air-fluid exchange [26]. But the toxic and uncertain effects of these additive on Müller cells and retinal pigment epithelium (RPE) cells remain unclear. Here, we inserted the inverted flap into the MH to stabilize the flap and injected NGF to improve healing process and we found that patients in both groups exhibited complete anatomical MH closure, from 1 to 6 months after surgery; the closure rates were thus 100%, implying NGF might be a synergetic protective factor during ILM insertion for the treatment of MHs.

ILM peeling allows residual Müller cells to reach the bare area of an MH, serving as a basement membrane regulating cell growth and as a scaffold for glial cell proliferation [10, 27, 28]. However, proliferation of activated glial cells triggers scarring; ILM fragments transplanted into holes may impede approximation of the neurosensory retina and the re-arranged photoreceptor cells [20]. We observed prominent foveal glial tissues on OCT scans of some eyes in both groups; although the MHs had closed, the BCVAs of these patients did not exhibit marked improvements. Park et al. indicated that no eye in an ILM insertion group exhibit complete recovery of the photoreceptor layers (the EZ and ELM) [20]. In this study, although only one eye of the control group and two of the NGF group exhibited complete recovery of ELM, and one of each group exhibited complete recovery of EZ integrity, the residual EZ and ELM defect sizes were significantly lower in the NGF group, and greater BCVA improvement and a shorter recovery period were observed. It is confirmed that a U-shaped closure is most common after use of the inverted-flap technique and is associated with better functional prognosis than other types of closure [23]. In our data, U-shaped foveae were observed in five of nine (55.6%) eyes in the NGF group and three of nine (33.3%) in the insertion group. We found no significant between-group difference in foveal configuration. Interestingly, the foveal configuration might be less important in terms of functional prognosis than hitherto thought, because several eyes in the NGF group with V-shaped or on the other hand not very good U-shaped foveae exhibited excellent EZ and ELM recoveries and good postoperative BCVAs (Fig. 4, other OCT scans data not show). Thus, NGF might aid photoreceptor recovery and improve visual acuity, although the detailed mechanisms require further elucidation.

NGF is a classical neuroprotective factor produced by Müller cells and plays a critical role in retinal neovascularization [18]. NGF can reduce photoreceptor apoptosis after retinal detachment injury and protect Müller cells, relieving rat retinal gliosis by modulating the Trk-A signaling pathway [29]. NGF also supports the existence of retinal ganglion cells (RGCs) and photoreceptors, which directly inhibits degeneration, and NGF stimulates additional neurotrophic factor expression from Müller cells, which indirectly enhances photoreceptor survival [30, 31]. Intravitreal NGF injection has been tested in the rat model of retinitis pigmentosa of the Royal College of Surgeons [32]. Liu and Ying et al. had suggested that 35 retinitis pigmentosa patients were treated with repeat intravitreal injections of NGF (30 μg/0.15 mL) (final concentration was about 6 μg/mL in the vitreous cavity) to protect the RPE after safety evaluation in rats and rabbits [33] and no toxic reactions or side-effects were evident. In this study, we employed the same NGF concentration, and found no toxic effects or abnormal tissue reactions during the entire follow-up. Our previous in vitro research also revealed that NGF was not toxic to Müller cells [34].

The limitations of our study included the small sample size and the relatively short follow-up period. The patients were limited because of the lack of C3F8 which was necessary for the MH surgery in our study. We had to stop our investigation when the C3F8 ran out. C3F8 production was banned in our country and C3F8 produced by foreign manufacturers had not been approved by NMPA. We sincerely hope that the production of C3F8 could be restored to clinical use as soon as possible. Our study was a very preliminary study and randomized controlled clinical studies involving a larger number of patients and longer follow-ups were required to evaluate the anatomical and functional outcomes of the central macular fovea and the safety of NGF. However, our findings suggested that NGF could be used to treat large MHs.

## Conclusions

In this study, we found that ILM sheet insertion into a MH combined with NGF injection had the potential to improve visual function in patients with large iFTMH in the short time for the initial surgical treatment. NGF produced better recovery of the photoreceptor layers and, consequently, might have superior postoperative visual acuity. Therefore, NGF could be valuable during initial treatment of large iFTMHs.

## Data Availability

The datasets used and analyzed during the current study are available from the corresponding author on reasonable request.

## Abbreviations

BCVA: Best-corrected visual acuity
ELM: External limiting membrane
EZ: Ellipsoid zone
FTMH: Full-thickness macular hole
ILM: Internal limiting membrane
MH: Macular hole
NGF: Nerve growth factor
NMPA: National Medical Products Administration
OCT: Optical coherence tomography
PPV: Pars plana vitrectomy
RCGs: Retinal ganglion cells
RPE: Retinal pigment epithelium
VEGF: Vascular endothelial growth factor
